# Metabolic Effects of Oral Phenelzine Treatment on High-Sucrose-Drinking Mice

**DOI:** 10.3390/ijms19102904

**Published:** 2018-09-25

**Authors:** Christian Carpéné, Saioa Gómez-Zorita, Alice Chaplin, Josep Mercader

**Affiliations:** 1 INSERM U1048, Institute of Metabolic and Cardiovascular Diseases (I2MC), University Paul Sabatier, 31059 Toulouse, France; 2 Nutrition and Obesity Group, Department of Nutrition and Food Science, University of the Basque Country (UPV/EHU) and Lucio Lascaray Research Institute, 48940 Vitoria, Spain; saioa.gomez@ehu.eus; 3 Biomedical Research Networking Centres, Physiopathology of Obesity and Nutrition (CIBERobn), Institute of Health Carlos III, 28029 Madrid, Spain; 4 Cardiovascular Research Institute, School of Medicine, Case Western Reserve University, Cleveland, OH 44106, USA; amc315@case.edu; 5 Laboratory of Molecular Biology, Nutrition and Biotechnology, University of the Balearic Islands, 07122 Palma, Spain; 6 Balearic Islands Health Research Institute (IdISBa), 07010 Palma, Spain

**Keywords:** phenelzine, obesity, adipocyte, lipogenesis, amine oxidases, hydrogen peroxide, hyperglycemia, oxidative stress

## Abstract

Phenelzine has been suggested to have an antiobesity effect by inhibiting de novo lipogenesis, which led us to investigate the metabolic effects of oral chronic phenelzine treatment in high-sucrose-drinking mice. Sucrose-drinking mice presented higher body weight gain and adiposity versus controls. Phenelzine addition did not decrease such parameters, even though fat pad lipid content and weights were not different from controls. In visceral adipocytes, phenelzine did not impair insulin-stimulated de novo lipogenesis and had no effect on lipolysis. However, phenelzine reduced the mRNA levels of glucose transporters 1 and 4 and phosphoenolpyruvate carboxykinase in inguinal white adipose tissue (iWAT), and altered circulating levels of free fatty acids (FFA) and glycerol. Interestingly, glycemia was restored in phenelzine-treated mice, which also had higher insulinaemia. Phenelzine-treated mice presented higher rectal temperature, which was associated to reduced mRNA levels of uncoupling protein 1 in brown adipose tissue. Furthermore, unlike sucrose-drinking mice, hepatic malondialdehyde levels were not altered. In conclusion, although de novo lipogenesis was not inhibited by phenelzine, the data suggest that the ability to re-esterify FFA is impaired in iWAT. Moreover, the effects on glucose homeostasis and oxidative stress suggest that phenelzine could alleviate obesity-related alterations and deserves further investigation in obesity models.

## 1. Introduction

Phenelzine is a drug prescribed for atypical depression, bipolar depression, and major depressive disorder that is resistant to other antidepressant drugs. Its mechanism of action relies on the irreversible and nonselective inhibition of both A and B isoforms of monoamine oxidase (MAO) [1]. MAO is a mitochondrial enzyme that catalyses the oxidative deamination of biogenic amines, dopamine, norepinephrine, and serotonin, as well as dietary amines. Consequently, inhibition of MAO increases the central levels of neurotransmitters [2,3]. MAO-A is expressed broadly, being particularly high in fat tissue, whilst MAO-B is highly expressed in the liver, among others. In addition to MAO inhibition, phenelzine potently inhibits semicarbazide-sensitive amine oxidase (SSAO), one of the proteins with the highest expression in adipocytes. SSAO, also known as vascular adhesion protein 1 (VAP-1), is a membrane-bound protein that oxidizes amines, producing aldehydes, ammonia, and hydrogen peroxide, and is codified by the amine oxidase copper-containing 3 (*Aoc3*) gene. In turn, SSAO/VAP-1 is expressed by vascular endothelial cells and facilitates the accumulation of inflammatory cells. SSAO inhibitors have emerged as interesting agents to combat obesity due to their ability to block the lipogenic effect of hydrogen peroxide [4]. Indeed, we have shown that adipose tissue mass is decreased after the administration of selective inhibitors of SSAO and MAO in rodents [5,6,7]. Phenelzine is also presented as a candidate molecule for reducing excess adiposity. In obese rodents, chronic administration of phenelzine modestly decreased the weight of visceral fat pads in female Zucker rats [7] and in male mice fed a high-fat diet [8]. The effect on adipose tissue enlargement is partly attributed to the inhibition of SSAO activity, since glucose uptake and de novo lipogenesis induced by benzylamine, a SSAO substrate, were inhibited by phenelzine in adipocytes [7,9]. Remarkably, insulin-stimulated de novo lipogenesis is also inhibited by phenelzine [9], indicating that mechanisms other than amine oxidase inhibition are involved in its antilipogenic effect. Indeed, another mechanism by which phenelzine inhibits the assembly of triacylglycerides is by targeting sterol regulatory element-binding protein-1c (SREBP-1c) in differentiating adipocytes. Such an effect eventually inhibits adipocyte differentiation, which has been shown in several murine cell lines and in human primary cultures of a stromal vascular fraction derived from adipose tissue and bone marrow mesenchymal stem cells [10,11]. Furthermore, we have recently shown that chronic phenelzine administration reduces body fat accumulation even in normal-weight mice, which was associated to a lower rate of insulin- and benzylamine-stimulated glucose incorporation into lipids in adipocytes [12]. Fat-reducing effects evidenced in animals and cultured cells contrasts with the weight gain observed in patients with mood disorders treated with this compound [13]. It remains unclear, however, whether it is the improvement of mood disorder or the drug itself that is involved in the weight gain following antidepressant therapy. Therefore, more studies are required in order to propose phenelzine as an antiobesity drug in humans, particularly in individuals without mood disorders. Another unwanted side effect of the antidepressant is the increase of hypertensive crisis when dietary tyramine intake is not restricted [14]. However, we recently described that body fat reduction was achieved without cardiovascular changes [12], reinforcing the potential benefit of phenelzine against obesity.

Whether phenelzine affects metabolic disturbances associated to obesity has not been explored so far. Such disturbances could be influenced by phenelzine not only secondary to its direct effect on adipose tissue, but also directly when considering the reported effects on glycemia homeostasis, inflammation, and oxidative stress. Indeed, the hypoglycemic effect of phenelzine is well known in depressed, normal-weight, nondiabetic individuals [15]. This could be explained by several mechanisms of action, including an inhibition of jejunal glucose uptake [16], an inhibition of hepatic gluconeogenesis [17], and an enhancement of glucose-mediated insulin release from the pancreas [18]. It therefore becomes interesting to determine whether phenelzine treatment can reduce hyperglycemia in animal models of obesity or diabetes. Increased inflammation and oxidative stress associated to obesity could be influenced by phenelzine, owing to its ability to inhibit both MAO and SSAO/VAP-1 action. By inhibiting the adhesion activity of VAP-1, phenelzine could influence the proinflammatory state observed in obesity [19]. On the other hand, by inhibiting hydrogen peroxide production, oxidative stress status associated to obesity could be reduced. Such effects could be relevant in adipose tissue given the high SSAO expression in adipocytes, and because both the expression and activity of SSAO are increased in obese and diabetic animals [20]. Furthermore, owing to its structure, hydrazine derivatives such as phenelzine display scavenging activity. They reduce oxidative stress induced by lipid peroxidation byproducts [21,22,23], prevent the formation of foam cells, and inhibit the formation of adducts between oxidized polyunsaturated fatty acids on free amino groups and thiol residues [24].

In this study, we exposed mice chronically to a high-sucrose solution in order to increase de novo lipogenesis and hence investigate the metabolic effects of phenelzine. Our results show that phenelzine did not exert a prominent impact on body adiposity, but restored glycemia and exerted interesting metabolic effects that deserve further studies on the putative impact of phenelzine or other MAO inhibitors on obesity, glucose and lipid homeostasis, and oxidative stress in animal models of obesity and diabetes.

## 2. Results

### 2.1. Oral Chronic Phenelzine Treatment Has No Effect on Body Weight Gain and Adiposity of Sucrose-Drinking Mice

**Figure 1:**
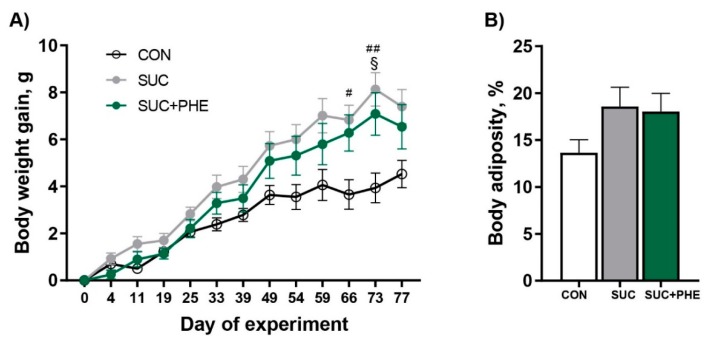
Influence of sucrose and phenelzine chronic treatments on body weight gain and adiposity of mice. Eleven-week-old C57BL/6 male mice received water (CON, in white), 20% sucrose solution (SUC, in grey), or sucrose solution plus 102 µmol phenelzine/kg body weight/day (SUC+PHE, in green) to drink for 11 weeks. (**A**) Cumulative body weight was recorded throughout the treatment. Data are the mean ± SEM of 8–12 animals per group; (**B**) Body adiposity was analysed by magnetic resonance imaging (MRI) at week 10. One-way ANOVA was followed by a Bonferroni post-hoc test. Difference CON vs. SUC mice: ^#^ *p* < 0.05; ^##^ *p* < 0.01; difference CON vs. SUC+PHE mice: ^§^ *p* < 0.05.

Initially, we analysed the influence of chronic exposure to a 20% sucrose drink on adiposity and body weight. For this, we recorded cumulative body weight and found that sucrose-drinking mice (SUC) gained more body weight, which was evident from day 66 (Figure 1A), and presented a greater body weight by the end of the experiment versus control water-drinking mice (CON) (Table 1). Body composition analysis via magnetic resonance imaging (MRI) performed one week before the end of the treatment showed, however, no significant differences. (Figure 1B). At the end of the treatment, the weights of liver, inguinal white adipose tissue (iWAT), and interscapular brown adipose tissue (iBAT) pads were heavier in SUC versus CON mice, when expressed as percentage of body weight. On the other hand, the weights of visceral WAT, epididymal (eWAT), and perirenal (pWAT) pads and the adiposomatic index remained unchanged. Surprisingly, when phenelzine was added to the sucrose drink (SUC + PHE), animals also gained more body weight than CON mice, which was evident from day 73 (Figure 1A). However, the impact of the sucrose treatment was lower in the presence of phenelzine, since the significantly increased iWAT and iBAT pad weights and body weight at sacrifice observed in SUC mice were not different from controls in the SUC + PHE group. However, when comparing SUC and SUC + PHE groups, we found no differences in body fat (Figure 1B), body weight, liver, or fat pad weights, or adiposomatic index (Table 1). There were also no differences regarding accumulated sucrose, chow, and energy intake at the end of the treatment or energy efficiency between SUC and SUC + PHE mice (Table 1). Importantly, circulating levels of transaminases were not altered in SUC + PHE mice (alanine aminotransferase (ALT): 24 ± 1 U/L; alkaline phosphatase (ALP): 19 ± 5 U/L), indicating that the treatment was not hepatotoxic.

We then went on to analyse the effect of sucrose exposure on the lipid content of eWAT and iWAT pads, as well as in liver and skeletal muscle tissue, since we have previously reported that phenelzine reduced ectopic fat in normal-weight mice [12] (Table 1). Total lipid content in eWAT and iWAT pads were increased in SUC versus CON mice, whereas the total protein content was unchanged. In addition, DNA content was also increased in the iWAT of SUC animals, suggesting that the number of cells could be increased in this fat pad. Although the ectopic lipid content in muscle and whole liver was not increased in SUC versus CON mice, the amount of hepatic triacylglycerides was 3.4-fold higher in SUC mice, as expected. Unlike SUC mice, lipid content in eWAT and iWAT of SUC + PHE mice was not different from the CON group. However, phenelzine addition to the sucrose drink did not normalize the parameters detailed above, as no significant differences were observed between SUC + PHE versus SUC mice.

### 2.2. Chronic Phenelzine Treatment Does Not Alter Lipogenic and Lipolytic Activities, but Reduces Pepck Expression in the iWAT of Sucrose-Drinking Mice

**Figure 2:**
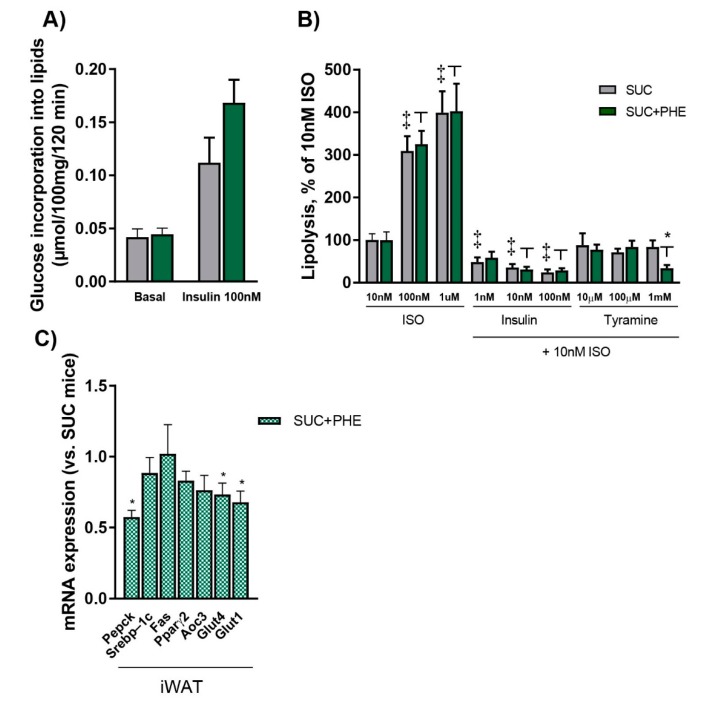
Effects of phenelzine treatment on lipogenesis and lipolysis, and the expression of genes related to adipocyte function in inguinal white adipose tissue (iWAT). Eleven-week-old C57BL/6 male mice received 20% sucrose solution (SUC, in grey), or sucrose solution plus 102 µmol phenelzine/kg body weight/day (SUC+PHE, in green) to drink for 11 weeks. (**A**) De novo lipogenesis: Murine white adipocytes were isolated from epididymal and perirenal WAT pads. Lipogenesis was measured as glucose incorporation into neosynthesized lipids after 120 min incubation with or without the indicated compounds. (**B**) Lipolysis: Murine white adipocytes were isolated from epididymal and perirenal WAT pads. Lipolysis was measured as glycerol released into the medium after 90 min incubation with or without the indicated compounds. ISO, isoprenaline. (**C**) mRNA levels in iWAT. mRNA levels are given relative to 18S rRNA/SUC mice, which are set to 1. Data are the mean ± SEM of 12 animals per group. A Student’s *t*-test was performed in all cases. Difference SUC vs. SUC+PHE: * *p* < 0.05; significant difference in lipolysis between treatments was considered *p* < 0.05 and is indicated as ‡ vs. SUC and as ┬ vs. SUC+PHE.

Since it has been previously shown that phenelzine impairs de novo lipogenesis pathway in isolated adipocytes [9,10], as well as in vivo after chronic phenelzine treatment [12], we assessed the incorporation of glucose into lipids to measure de novo lipogenesis in visceral white adipocytes from SUC and SUC + PHE mice. Basal de novo lipogenesis was unchanged in SUC + PHE compared to SUC mice (Figure 2A). Furthermore, there were also no differences in de novo lipogenesis stimulated by 100 nM insulin plus 100 nM vanadate, although a trend to a higher rate was observed in SUC + PHE mice (*p* = 0.094), or by benzylamine plus 100 nM vanadate (not shown), suggesting that de novo lipogenesis is not impaired by phenelzine in sucrose-drinking mice.

We also checked whether there were differences in the lipolytic activity of isoprenaline and the antilipolytic action of insulin and tyramine between SUC and SUC + PHE mice. Lipolysis was measured as the glycerol was released into the medium in response to isoprenaline. We observed that the lipolytic effect of increasing doses of isoprenaline from 10 nM to 1 µM was identical in adipocytes from SUC and SUC + PHE mice. Moreover, the antilipolytic action of increasing concentrations of insulin from 1 nM to 100 nM was observed in SUC mice; however, in SUC + PHE mice, the action of insulin was only observed from 10 nM to 100 nM, but not at 1 nM. Tyramine concentrations ranging from 10 µM to 1 mM did not elicit an antilipolytic effect in SUC mice, whereas in phenelzine-receiving mice, the effect of tyramine was observed at 1 mM (Figure 2B).

We further explored the potential effects of phenelzine on adipose tissue by analysing the mRNA expression of key genes in fatty acid re-esterification, glucose uptake, lipogenesis, and adipocyte differentiation in the subcutaneous iWAT from SUC and SUC + PHE mice. First, we analysed the expression of phosphoenolpyruvate carboxykinase (*Pepck*), a key gene in fatty acid re-esterification, which has been previously shown to be reduced in iWAT of normal-weight mice chronically treated with phenelzine [12]. In SUC + PHE mice, *Pepck* expression was reduced by 43% compared to the SUC group, suggesting that fatty acid re-esterification could be a potential target of the drug. We next measured the expression of the transcription factor *Srebp*-*1c*, considering that it has been previously shown to be a target of phenelzine in cultured adipocytes [10]. The expression of *Srebp*-*1c* and its target gene, fatty acid synthase (*Fas*), which are involved in de novo lipogenesis, were unchanged in sucrose-drinking mice. Furthermore, peroxisome proliferator-activated receptor gamma 2 (*Pparγ2*)*,* the master regulator of adipocyte differentiation, and *Aoc3*, a marker of adipocyte terminal differentiation, were not changed in SUC + PHE versus SUC mice. Both glucose transporter 4 (*Glut4*) and glucose transporter 1 (*Glut1*) were reduced by 27% and 32%, respectively, in animals treated with phenelzine, suggesting that the ability to uptake glucose could be impaired in iWAT (Figure 2C).

### 2.3. Impact of Phenelzine Treatment on Circulating Parameters Related to Lipid Metabolism in Sucrose-Drinking Mice

**Figure 3:**
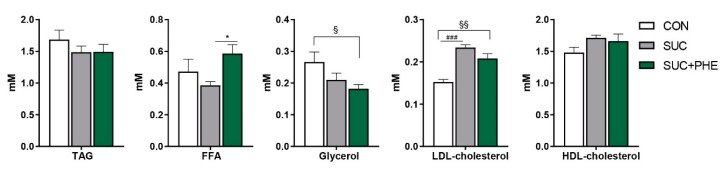
Influence of sucrose and phenelzine chronic treatments on circulating lipid-related parameters in mice. Eleven-week-old C57BL/6 male mice received water (CON, in white), 20% sucrose solution (SUC, in grey), or sucrose solution plus 102 µmol phenelzine/kg body weight/day (SUC + PHE, in green) to drink for 11 weeks. Data are the mean ± SEM of 8–12 animals per group. TAG, triacylglycerols; FFA, free fatty acids; LDL, low-density lipoprotein; HDL, high-density lipoprotein. One-way ANOVA was followed by a Bonferroni post-hoc test. Difference CON vs. SUC mice: ^###^ *p* < 0.001; difference CON vs. SUC+PHE mice: ^§^ *p* < 0.05; ^§§^ *p* < 0.01; difference SUC vs. SUC+PHE: * *p* < 0.05.

It was of our interest to see whether changes in body weight were accompanied by alterations in the main circulating parameters related to lipid metabolism in SUC mice, and to study the potential impact of phenelzine. Circulating levels of triacylglycerol, free fatty acids (FFA), glycerol, and high-density lipoprotein (HDL) cholesterol were unaltered in SUC mice. Plasma low-density lipoprotein (LDL) cholesterol levels were increased by 53% in SUC mice. Interestingly, PHE + SUC mice presented no differences regarding plasma triacylglycerol, HDL-cholesterol, and FFA levels when compared to control animals. However, circulating FFA levels were higher in SUC + PHE versus SUC mice, suggesting higher lipolysis or lower fatty acid re-esterification. Furthermore, plasma glycerol levels of SUC + PHE mice were not different from SUC mice, but were lower than in CON mice. Lastly, plasma LDL-cholesterol levels were increased in SUC + PHE mice, but to a lower extent (36% increase) than in SUC mice. However, the difference in LDL-cholesterol levels between SUC + PHE versus SUC mice was not significant (*p =* 0.119) (Figure 3).

### 2.4. Phenelzine Treatment Restores Fasting Glycemia at the Expense of Increasing Insulinemia in Sucrose-Drinking Mice 

**Figure 4:**
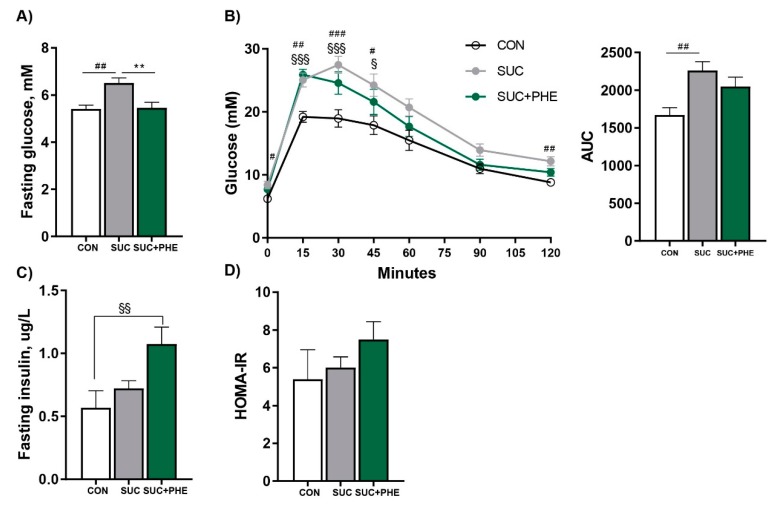
Influence of sucrose and phenelzine chronic treatments on glucose and insulin homeostasis. Eleven-week-old C57BL/6 male mice received water (CON, in white), 20% sucrose solution (SUC, in grey), or sucrose solution plus 102 µmol phenelzine/kg body weight/day (SUC+PHE, in green) to drink for 11 weeks. (**A**) Fasting blood glucose levels at week 9. (**B**) Blood glucose levels after an oral glucose tolerance test at week 10. AUC, area under the curve. (**C**) Fasting blood insulin levels at week 9. (**D**) Homeostatic model assessment for insulin resistance (HOMA-IR) score. Data are the mean ± SEM of 8–12 animals per group. One-way ANOVA was followed by a Bonferroni post-hoc test. Difference CON vs. SUC mice: ^#^ *p* < 0.05; ^##^ *p* < 0.01; ^###^ *p* < 0.001; difference CON vs. SUC+PHE mice: ^§^ *p* < 0.05; ^§§^ *p* < 0.01; ^§§§^ *p* < 0.001; difference SUC vs. SUC+PHE: ** *p* < 0.01.

Nonfasting blood glucose was analysed once a week and levels did not differ between the three groups (CON: 9.45 ± 0.10 mM; SUC: 9.73 ± 0.10 mM; SUC + PHE: 9.79 ± 0.11 mM; mean values of 11 determinations, non-significant). An impaired glucose homeostasis elicited by sucrose treatment was confirmed at week 10, when SUC mice presented higher fasting blood glucose levels than CON animals. Interestingly, phenelzine had a potentially beneficial effect on glucose control in sucrose-drinking mice, since it reduced fasting glycemia to controls levels, evidencing its hypoglycemic effect even in mice receiving a chronic sucrose intake (Figure 4A).

To further explore whether phenelzine treatment induced a better response to glucose overload, an oral glucose tolerance test was performed. Glucose intolerance was demonstrated in SUC mice since they displayed higher glucose levels at 0, 15, 30, 45 and 120 min of the oral glucose tolerance test and a higher area under the curve (AUC) than CON mice. SUC + PHE mice also displayed higher glucose levels than CON mice at 15, 30 and 45 min, but not thereafter, and the AUC was not different from the CON group. No differences were observed between the AUCs of SUC and SUC + PHE mice (Figure 4B). Fasting insulin levels (Figure 4C) and the homeostatic model assessment for insulin resistance (HOMA-IR) (Figure 4D) values were normal in SUC mice, indicating that these animals did not develop insulin resistance. Surprisingly, insulin levels in SUC + PHE mice were higher than both SUC and CON mice. HOMA-IR values suggested, however, that SUC + PHE mice might not be insulin-resistant, since there were no differences among groups. Given the effects of sucrose and phenelzine treatment on fasting glycemia, we decided to analyse the levels of plasma fructosamine, which is considered a biomarker of hyperglycemia. Fructosamine levels for the CON, SUC, and SUC + PHE groups were, respectively, 211 ± 5 µM, 225 ± 5 µM, and 209 ± 8 µM, with no statistical differences among groups.

### 2.5. Phenelzine Treatment Increases Rectal Temperature, but Appears to Be Unrelated to BAT Thermogenic and Muscle Catabolic Capacity

**Figure 5:**
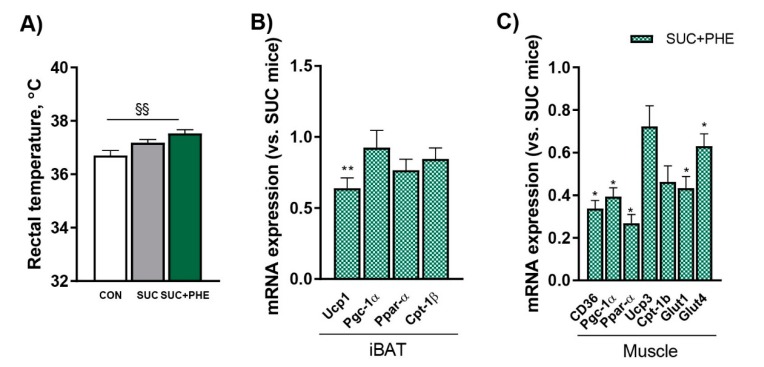
Effects of phenelzine treatment on rectal temperature and the expression of genes related to thermogenesis, lipid handling and catabolism, and glucose uptake in interscapular brown adipose tissue (iBAT) and skeletal muscle. Eleven-week-old C57BL/6 male mice received water (CON, in white), 20% sucrose solution (SUC, in grey), or sucrose solution plus 102 µmol phenelzine/kg body weight/day (SUC+PHE, in green) to drink for 11 weeks. (**A**) Rectal temperature was assessed at 3 time points during the treatment. (**B**) mRNA levels in iBAT. (**C**) mRNA levels in skeletal muscle. mRNA levels are given relative to 18S rRNA/SUC mice, which are set to 1. When comparing all three groups, a one-way ANOVA followed by a Bonferroni post-hoc test was used; when comparing SUC vs. SUC + PHE (**B**), a Student’s *t*-test was performed. Difference CON vs. SUC+PHE mice: ^§§^ *p* < 0.01; difference SUC vs. SUC+PHE: * *p* < 0.05; ** *p* < 0.01.

Since the administration of diets with a relatively high carbohydrate content increases body temperature in mammals [25,26] and the administration of a MAO inhibitor potentiates the hyperthermic effect of amines [27], we wondered whether phenelzine could influence body temperature in sucrose-drinking mice. Rectal temperature was assessed at three time points during the treatment and the mean value is presented in Figure 5A. Compared to CON mice, a clear trend towards increased rectal temperature was observed in SUC mice (*p =* 0.094), whilst SUC + PHE mice showed a significant higher rectal temperature.

To study whether the increase in rectal temperature observed in phenelzine-treated animals was associated to higher BAT thermogenesis and oxidative metabolism capacities, we analysed the expression of key genes in these processes in the iBAT and muscle of SUC and SUC + PHE mice. Interestingly, we observed that the mRNA levels of uncoupling protein 1 (*Ucp1*) in iBAT were decreased by 36% in SUC + PHE versus SUC mice; however, when looking at genes involved in fatty acid oxidation, including peroxisome proliferator-activated receptor coactivator gamma 1α (*Pgc*-*1α*), peroxisome proliferator activated receptor alpha (*Pparα*), and carnitine palmitoyl transferase 1b (*Cpt*-*1b*), no significant differences were found (Figure 5B). In view of these findings, it seems plausible to consider that UCP1-dependent thermogenesis does not contribute to the enhanced increase in rectal temperature observed in SUC + PHE mice.

When looking at muscle, it was interesting to observe that the mRNA levels of genes with a pivotal role in fatty acid uptake and catabolism were reduced in SUC + PHE mice, as it was the case for cluster of differentiation 36 (*Cd36*, by 67%), *Pgc*-*1α* (by 51%), and peroxisome proliferator-activated receptor alpha (*Pparα*, by 80%), or tended to be lower, in the case of *Cpt*-*1b (p =* 0.051*)*. The mRNA levels of uncoupling protein 3 (*Ucp3*) were not changed. The mRNA levels of *Glut1* and *Glut4*, which are involved in, respectively, nondependent and insulin-dependent glucose uptake, were reduced by 46% and 24% in SUC + PHE versus SUC mice (Figure 5C). Collectively, this data suggests a lower capacity for glucose and lipid uptake and for lipid catabolism in SUC + PHE mice.

### 2.6. Effects of Phenelzine Treatment on Indicators of Oxidative Stress and Inflammation

**Figure 6:**
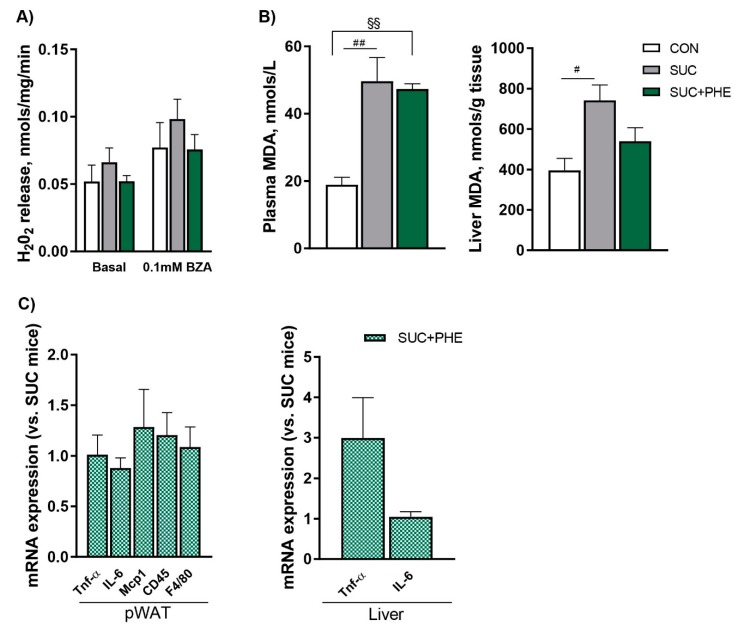
Effects of sucrose and phenelzine treatments on oxidative stress markers. Eleven-week-old C57BL/6 male mice received water (CON, in white), 20% sucrose solution (SUC, in grey), or sucrose solution plus 102 µmol phenelzine/kg body weight/day (SUC+PHE, in green) to drink for 11 weeks. (**A**) Hydrogen peroxide release in inguinal WAT. Fresh intact tissue (30 mg) was incubated without or with 0.1 mM benzylamine (BZA). (**B**) Lipid peroxidation was measured as malondialdehyde (MDA) levels. Data are the mean ± SEM of 8 (CON), 12 (SUC), and 12 (SUC+PHE) animals per group. (**C**) mRNA levels in perirenal WAT and liver. mRNA levels are given relative to 18S rRNA/SUC mice. One-way ANOVA was followed by a Bonferroni post-hoc test. Difference CON vs. SUC mice: ^#^ *p* < 0.05; ^##^ *p* < 0.01; difference CON vs. SUC+PHE mice: ^§§^ *p* < 0.01.

Hydrogen peroxide release was measured in fresh intact iWAT pieces in basal conditions and after incubating the tissue with 0.1 mM benzylamine. There were no differences regarding basal or 0.1 mM benzylamine-stimulated hydrogen peroxide release between the three groups (Figure 6A). Next, we assessed lipid peroxidation by measuring malondialdehyde levels in plasma and liver samples. In comparison to controls, SUC mice presented increased levels of malondialdehyde both in plasma and liver. SUC + PHE mice also presented higher malondialdehyde levels in plasma versus controls. Hepatic malondialdehyde levels, however, were not different from CON mice, and when compared to SUC mice, the difference was not significant (*p =* 0.125) (Figure 6B). Lastly, we measured the mRNA levels of the proinflammatory cytokines tumor necrosis factor α (*Tnf-α*) and interleukin-6 (*Il-6*) in pWAT and liver. We also measured the mRNA levels of the markers of immune cell infiltration monocyte chemoattractant protein 1 (*Mcp1*), *Cd45*, and *F4/80* in pWAT. Phenelzine treatment did not change the mRNA levels of any of the inflammation-related genes (Figure 6C).

## 3. Discussion

In this study, we aimed to investigate the effects of phenelzine on adiposity and several obesity-related metabolic disturbances. Taking into account that it has been previously shown to impair de novo lipogenesis in adipocytes, we hypothesised that phenelzine could limit adipose tissue development and body weight when it was chronically administrated in animals, in which de novo lipogenesis is expected to be activated. For this purpose, we exposed mice to a high-sucrose solution to drink. Up until now, the described metabolic-related effects of a chronic exposure to sucrose solutions largely depend on sucrose concentration, species, sex, and strain. Increased hepatic triacylglycerol content, adiposity and body weight gain, hyperglycemia, hyperinsulinemia, and hypercholesterolemia are all alterations that can be developed when giving 10% to 30% sucrose concentrations to rats and mice [28,29,30,31], and the de novo lipogenesis rate in adipose tissue is increased by a 20% sucrose solution in rats [32]. In our experiment, we found that chronic sucrose drinking had a modest effect on fat accumulation in adipose tissue of male C57BL/6 mice. Furthermore, by the end of the study, animals presented an increase in body weight gain versus controls, which could be partly attributed to the higher weights of subcutaneous fat pads (iWAT and iBAT) and livers. This was consistent with the higher contents of lipids and DNA in iWAT and triacylglycerols in the liver. However, despite an increase in total lipid content in eWAT, sucrose did not dramatically extend the mass of the dissected visceral pads.

Even though the increase in fat accumulation elicited by sucrose was small, phenelzine treatment was expected to limit adipose tissue enlargement as it did in rodent obesity models in which lipid accumulation is prominent in fat deposits and liver, namely in Zucker rats and mice fed a very high-fat diet [7,8]. In the present study, in which phenelzine is orally administrated, it is unlikely that the lack of effect on fat accumulation in sucrose-drinking mice can be attributed to a limited obesogenic challenge, since phenelzine has been reported to affect adiposity even in mice kept under standard conditions [12]. Taking the results from chronic oral treatments together, it is suggested that the different effects of phenelzine on fat pad mass could depend on diet. It is unlikely that the milder effect on fat accumulation in sucrose-drinking mice can be attributed to the phenelzine dose. When comparing the mean daily doses that sucrose-drinking mice received with previous experiments in which phenelzine was also given orally to mice, we noted that sucrose-drinking mice received a dose (102 ± 10 µmol/kg) comprising between that received by normal-weight (135 ± 6 µmol/kg) and obese (68 µmol/kg) mice. Nevertheless, the dose used in this experiment may have not been sufficient to completely block the activities of MAO and SSAO in adipose tissue, which could not be measured and is a limitation of the present study. Incomplete SSAO inhibition could be suggested by the lack of differences in hydrogen peroxide production between SUC and SUC + PHE mice, especially in response to benzylamine (BZA), which was able to activate the lipogenic process in in previous experiments [7,9,12]. On the other hand, the antilipolytic effect of tyramine was evidenced only in phenelzine-treated mice and was not at all indicative of the extent of the SSAO and/or MAO inhibition. Regardless of MAO/SSAO activities, other phenelzine targets involved in its purported antilipogenic activity, such as the inhibition of *Srebp*-*1c* seen in cultured adipocytes [10], seem to not be involved in SUC mice, since the mRNA levels of *Srebp*-*1c* and its target genes were not reduced in iWAT pads. This result was also observed in our previous experiment using normal-weight mice [12], suggesting that oral phenelzine does not regulate *Srebp*-*1c* expression in vivo*.* Furthermore, phenelzine has been reported to inhibit insulin-stimulated glucose incorporation into lipids, both when directly administrated in vitro on adipocyte suspensions [9] and in adipocytes from phenelzine-treated mice [12]. Such an inhibitory effect was not seen in sucrose-drinking mice, suggesting that sucrose administration induces an adaptation that surpasses the antilipogenic effect of phenelzine and/or that phenelzine plus sucrose triggers other mechanisms in adipocytes that counteract the antilipogenic effect of the drug. Counteracting antilipogenic phenelzine actions could involve SSAO-dependent and SSAO-independent mechanisms. Accordingly, we explored other potential phenelzine targets by analysing gene expression in iWAT. These data revealed that rather than counteracting the antilipogenic action of phenelzine, they point to a potential inhibition of fat accumulation by targeting the expression of genes consistent with an antifattening effect. It is thus proposed that the ability to uptake glucose and re-esterify triacylglycerols could be impaired, since the mRNA expression levels of *Glut4*, *Glut1* and *Pepck* were decreased, which in turn, would be in line with the biometric parameters measured in iWAT. Indeed, in the iWAT pad of SUC + PHE mice, fat accumulation does not seem to be as altered as in SUC mice, given the lack of differences in iWAT total lipid content and mass between SUC + PHE and CON animals. The potential effect of phenelzine on adipose tissue triacylglycerol re-esterification is in agreement with previous results [12] and is consistent with the higher circulating FFA levels found in SUC + PHE mice, which, on the other hand, would be unlikely to come from an enhanced lipolytic rate, as there were no differences in the response to isoprenaline. The higher circulating FFA levels, together with the lower increase in circulating LDL-cholesterol levels seen in SUC + PHE mice, suggest that lipid homeostasis is influenced by phenelzine treatment. The effect on LDL-cholesterol is to be noted and contrasts with the altered lipid homeostasis observed in patients treated with antidepressants, which is commonly associated with weight gain [33].

Pepck is a key enzyme in hepatic gluconeogenesis, the activity of which is inhibited by phenelzine in the liver [34] and in adipose tissue ([12] and in the present study), and which undoubtedly contributes to the hypoglycemic effect of the drug. According to the literature, hypoglycaemia can also be explained by an inhibition of glucose uptake in the jejunum and an increase in insulin secretion [16,18]. The lowering effect of phenelzine in fasting glycemia could be explained by an inhibition of hepatic gluconeogenesis, which is activated in fasting conditions. However, an action of phenelzine on jejunum glucose uptake is not expected to be relevant in SUC + PHE mice, according to the levels of nonfasting glucose, which were not different from sucrose-drinking mice. In addition to decreased gluconeogenesis, increased plasma insulin levels elicited by phenelzine treatment could be in part responsible for the lower fasting glucose levels. Circulating glucose, in turn, may be taken up more efficiently by insulin-dependent tissues of phenelzine-treated mice, including visceral adipocytes, potentially contributing to overcoming the antilipogenic effect of phenelzine. Phenelzine is known to affect insulin secretion, potentiating glucose-mediated insulin release from pancreas segments by mechanisms that are both MAO-dependent and -independent [18]. On the contrary, at millimolar concentrations, which are not reached in our experimental animals, it seems to inhibit glucose-mediated insulin release [35]. Unlike SUC + PHE mice, normal-weight mice treated with phenelzine maintain normal levels of insulin and glucose [12]. However, in mice continuously exposed to high glucose levels coming from the diet, phenelzine could have potentiated glucose-stimulated insulin release. It is therefore interesting to investigate whether phenelzine can normalize hyperglycemia in other models of obesity and diabetes.

The sympathetic system is expected to be activated in iBAT after chronic phenelzine treatment. We speculated that phenelzine could have potentiated the diet-induced thermogenic effect of sucrose treatment and, in fact, we observed that in sucrose-drinking mice, phenelzine treatment produced a stronger effect on rectal temperature than sucrose alone. However, the increase in temperature of phenelzine-treated animals may be attributed to the central actions of brain catecholamine levels, which are increased by the drug [2,3], rather than the peripheral actions of these catecholamines, because the expression of *Ucp1* in iBAT was not increased. On the contrary, *Ucp1* expression was decreased, and moreover, the mRNA expression of genes related to BAT activation was unchanged, suggesting that BAT thermogenesis was not related to the increased rectal temperature and does not seem to have a major impact on the potential effect of phenelzine on energy metabolism. Likewise, an activation of the sympathetic system produced by phenelzine treatment could have been expected in skeletal muscle, a tissue in which the role of MAO inhibition is largely unknown. However, an enhanced capacity for fuel oxidation in skeletal muscle can be ruled out, since the mRNA expression of genes related to glucose and fatty acid uptake and fatty acid catabolism (*Glut4*, *Glut1*, *Cd36*, *Pgc*-*1α* and *Pparα*) were all downregulated. On the contrary, these results rather suggest a decreased capacity for fuel handling and catabolism in sucrose-drinking mice, which could have contributed to counteract the potential effect of phenelzine on body adiposity. Unfortunately, gene expression analysis was performed only in SUC and SUC + PHE mice, and thus mRNA levels cannot be compared to the CON group, which represents a limitation of the experimental design.

Lastly, we assessed two indicators of oxidative stress, namely hydrogen peroxide production and levels of malondialdehyde, and the expression of genes related to inflammation. We hypothesized that phenelzine reduces oxidative stress in metabolic disorders not only owing to its ability to potently inhibit the hydrogen peroxide produced by SSAO and MAO, but also according to its reported effects as a reactive oxygen species (ROS) scavenger [21,22,23,24]. In fact, MAO inhibitors attenuated ROS production in the aorta and heart in a diabetic rat model [36]. Moreover, low-grade inflammation could be reduced, not only considering the impact on oxidative stress, but also given the inhibition of VAP-1 adhesion activity [19]. Sucrose-drinking mice did not present higher hydrogen peroxide levels in iWAT than control animals, with phenelzine having no significant effect on them either. The expression of proinflammatory cytokines and immune cell infiltration markers in pWAT were also not reduced by the drug. It can be argued that the increase in adipose fat accumulation elicited by the sucrose drink, which was actually not evidenced in pWAT fat pads, could not be sufficient to develop a proinflammatory state in adipose tissue. Therefore, it would be worth assessing its effect on adipose hydrogen peroxide production and inflammation in models of obesity in which such parameters are markedly altered. We then focused on the liver to study potential antioxidant and anti-inflammatory effects, since this organ is expected to be particularly affected by a chronic sucrose intake in terms of triacylglycerol accumulation [28,29,30]. Consistently, the livers of sucrose-drinking mice were heavier and presented elevated triacylglycerol and malondialdehyde levels. When phenelzine was administered, no effect on triacylglycerol content and liver mass was observed, which suggests that the drug does not have a major effect on de novo lipogenesis in hepatocytes of sucrose-drinking mice. Proinflammatory cytokine expression was not changed, and lipid peroxidation was not as altered as in SUC mice. The latter result suggests the potential antioxidative stress role of phenelzine in metabolic diseases, which might not occur in other body sites of sucrose-drinking mice, since it did not influence plasma malondialdehyde levels. Both potential antioxidant and anti-inflammatory effects of phenelzine deserve further studies in animal models of obesity.

## 4. Materials and Methods

### 4.1. Animals, Diets, and Treatments

C57BL/6 mice (Charles River, L’Arbresle, France) were housed under a 12/12 h light/dark cycle with controlled temperature (22 ± 2 °C) and humidity (50–60%), placing four animals per cage. Animal procedures performed complied with the principles established by the Institut National de la Santé et de la Recherche Médicale (INSERM, Toulouse, France) (Permission Number: 12-1048-03-15, on the 20 March 2012) and were approved by the Animal Ethics Committee of the unit US006 CREFRE (Centre Régional d’Exploration Fonctionnelle et Ressources Expérimentales, Toulouse, France).

Eleven-week-old mice of equivalent body weight were fed standard chow (Harlan, France). Animals were given either water to drink (CON, control mice, *n* = 8), a 20% sucrose solution (SUC, sucrose-drinking mice, *n* = 12), or phenelzine sulfate at an initial 0.028% (1.20 mM) concentration dissolved in a 20% sucrose solution (SUC + PHE, phenelzine-treated mice, *n* = 12). All mice had ad libitum access to chow and water/treatment solutions. Body weight and chow consumption was recorded weekly, and water drinking was determined twice a week. The duration of the treatment was 11 weeks. After the first week of treatment, we observed that the high-sucrose beverage increased water intake, and hence the amount of phenelzine was recalculated to achieve a dose within the range used in previous chronic experiments in which a reduction in adiposity was observed [8,12]. At the end of the experiment, the average phenelzine intake was 23 mg/kg body weight/day (102.2 ± 9.7 µM/kg body weight/day). This dose would correspond to 2 mg/kg in human subjects when using interspecies body surface area normalization [37]. Moreover, since PHE + SUC mice drank less compared to the SUC group after the third week of treatment, the amount of sucrose was from then on adjusted to ensure that both groups received the same amount.

### 4.2. In Vivo Determinations and Sacrifice

Body composition was analysed by MRI with the Echo MRI 100TM device (100TM3; Echo Medical Systems, Houston, TX, USA) at week 10. Nonfasting blood glucose was determined weekly between 15:00 and 16:00 h with a glucose monitor (Roche Diagnostics, Mannheim, Germany) using a blood drop withdrawn from the mouse tail. Fasting glucose was determined at week 9. An oral glucose tolerance test was performed on 8-h fasted mice at week 9. A glucose load of 3 g/kg body weight was given by oral gavage (time 0), and glucose levels were measured at time −30, 0, 15, 30, 45, 60, 90, and 120 min. HOMA-IR index was calculated as = (fasting plasma glucose) × (fasting serum insulin)/22.5, as previously described [38].

Mice were sacrificed by cervical dislocation after overnight fasting. Troncular blood was collected in heparinised tubes and processed to obtain plasma samples. Organ and tissue samples were immediately isolated and weighed. Tissue samples used for determining mRNA abundance and tissue composition were snap-frozen in liquid nitrogen and stored at −80 °C. Adipose tissue samples to determine lipolysis and hydrogen peroxide release were collected and immediately processed as described below.

### 4.3. Circulating Parameters and Tissue Composition Analysis

Circulating levels of HDL-cholesterol, LDL-cholesterol, triglycerides, free fatty acids, ALP, ALT, and fructosamine were determined with an autoanalyzer (Cobas Roche Diagnostic, Basel, Switzerland). Insulin was assayed with an ultrasensitive mouse ELISA kit (Mercodia, Uppsala, Sweden). Serum glycerol and hepatic triacylglycerol were determined by using enzymatic colorimetric kits (Sigma–Aldrich, Saint-Quentin-Fallavier, France). Organ lipid content was determined according to Dole and Meinertz [39]. Protein content was determined with the BCA protein assay kit (Pierce, Rockford, IL, USA). DNA content was determined with a fluorometric method.

### 4.4. Lipogenesis and Lipolysis

Adipocytes were isolated and digested by 15 µg/mL type TM liberase (Roche Diagnostics, Meylan, France) at 37 °C of epididymal and perirenal adipose tissues in Krebs–Ringer buffer pH 7.4, containing 15 mM bicarbonate, 10 mM HEPES, 5.5 mM glucose, and 3.5% of bovine serum albumin (KRBH buffer). Digestion was followed by filtration of the buoyant adipocytes with pieces of nylon stockings, and two washes in the same incubation buffer without liberase. Lipolytic activity was assessed by glycerol release as previously reported [6]. Briefly, adipocytes were diluted 10× in KRBH buffer. Samples at a final volume of 400 µL were incubated for 90 min with isoprenaline in the absence or presence of insulin and tyramine. Glycerol released was referred to 100 mg of cellular lipids and expressed as percentage of 10^−8^ M isoprenaline stimulation.

Lipogenic activity was determined by measuring the radioactivity incorporated from D– [3–^3^H] –glucose (Perkin Elmer, Waltham, MA, USA) into cellular lipids. Briefly, slight adaptations of the original radiometric insulin bioassay, detailed in [12], allowed us to determine the incorporation of 0.6 mM radioactive glucose into lipids during 120 min incubation. The same vials were used for lipid extraction in an organic mixture and counting of the labelled neosynthesized lipids in a non-water-miscible liquid scintillation cocktail (InstaFluor–Plus, PerkinElmer).

### 4.5. Gene Expression

Total RNA was extracted using column affinity purification (Qiagen, Courtaboeuf, France) according to the manufacturer’s instructions. Complementary DNA was synthetized using Superscript II reverse transcriptase (Life Technologies Corporation, Carlsbad, CA, USA) and random hexamers. Real-time PCR was performed in a Step One Plus system (Applied Biosystems, Warrington, UK), using the SYBR Green MasterMix (Eurogentec, Liège, Belgium). Oligonucleotide primers were designed and used as previously reported [40] and sequences are available upon request. Relative gene expression was calculated using the 2^ΔΔCt^ method with 18S ribosomal RNA as an internal control.

### 4.6. Oxidative Stress Biomarkers

Portions of 30 mg of intact fresh iWAT pads were used to assess hydrogen peroxide release. Tissue explants were incubated in KRBH buffer at 37 °C for 30 min in the absence or presence of 0.1 mM benzylamine. Hydrogen peroxide content was measured following the Amplex Red-based fluorometric method with slight modifications [41]. The chromogenic mixture was prepared by mixing 40 µM fluorescent substrate 10-acetyl-3,7 dihydrophenoxazine solution (Interchim, Montluçon, France) with 4 U/mL horseradish peroxidase in phosphate buffer pH 7.5. Fluorescence was detected on a Fluoroskan Ascent microplate reader (ThermoLabsystems, Vantaa, Finland). Hydrogen peroxide release was normalised per milligram of protein.

Lipid peroxidation was determined by measuring the formation of thiobarbituric acid reactive substrates TBARS (TBARS Assay Kit; Cayman Chemical Company, Ann Arbor, MI, USA).

### 4.7. Statistical Analysis

Experimental data are given as mean ± SEM of the indicated number of independent observations and were analysed by a one-way ANOVA followed by a Bonferroni post-hoc test (when comparing all three groups) and a Student’s *t*-test when comparing only two groups. Statistical significance was assumed when *p* < 0.05. IBM SPSS Statistics, version 25.0 (IBM Corp, Armonk, NY, USA) was used for statistical analyses and GraphPad Prism 7 was used to represent all data.

## 5. Conclusions

To conclude, our results suggest that the proposed antiobesity role of phenelzine is less relevant in high-sucrose-drinking mice. However, this work opens up a path to investigate novel applications of phenelzine on obesity-related metabolic pathologies, particularly as an antihyperglycemic agent, a modulator of lipid homeostasis, and as a potential oxidative stress scavenger in the context of metabolic diseases. Additionally, our work provides novel findings on the molecular targets of phenelzine in energy metabolism.

## Figures and Tables

**Table 1:** Influence of sucrose and phenelzine chronic treatments on biometric and tissue composition parameters in mice.

Parameter	CON	SUC	SUC + PHE	*p* Value
Body weight at baseline (g)	28.3 ± 1.2	28.2 ± 0.6	28.2 ± 0.8	
Body weight at week 11 (g)	32.6 ± 1.4	36.1 ± 0.7 #	34.1 ± 0.6	0.032
Ac. chow intake (g)		238 ± 17	231 ± 8	
Ac. sucrose intake (g)		37.5 ± 1.2	32.1 ± 2.5	
Ac. energy intake (kcal)		910 ± 54	867 ± 35	
Energy efficiency (g/Mcal)		8.5 ± 1.3	8.3 ± 3	
Adiposomatic index (%)	6.0 ± 0.5	8.0 ± 0.7	7.2 ± 0.7	
pWAT				
mass (%)	1.0 ± 0.1	1.3 ± 0.1	1.1 ± 0.1	
eWAT				
mass (%)	3.0 ± 0.2	3.6 ± 0.3	3.2 ± 0.3	
lipid (mg)	692 ± 83	1166 ± 119 ^#^	996 ± 105	0.023
protein (mg)	10.2 ± 1.4	10.2 ± 0.4	9.5 ± 1.6	
DNA (µg)	46.4 ± 5.4	40.8 ± 4.6	33.3 ± 2.9	
iWAT				
mass (%)	1.9 ± 0.1	2.8 ± 0.2 ^#^	2.4 ± 0.2	0.043
lipid (mg)	431 ± 51	782 ± 89 ^#^	676 ± 89	0.031
protein (mg)	9.3 ± 1.5	11.4 ± 1.3	12.9 ± 1.8	
DNA (µg)	35.1 ± 3.0	58.2 ± 4.9 ^#^	63.1 ± 6.4 ^§§^	0.003
iBAT				
mass (%)	0.31 ± 0.03	0.51 ± 0.04 ^#^	0.41 ± 0.04	0.032
Liver				
mass (%)	4.2 ± 0.3	4.8 ± 0.1 ^#^	4.8 ± 0.02 ^§^	0.013
lipid (mg)	51.6 ± 6.7	57.2 ± 11.0	66.7 ± 8.3	
protein (mg)	248 ± 2	277 ± 15	247 ± 12	
DNA (µg)	2177 ± 211	2088 ± 132	1803 ± 84	
TAG (mg/g tissue)	0.050 ± 0.003	0.168 ± 0.021 ^#^	0.175 ± 0.019 ^§§^	0.008
Skeletal muscle				
lipid (mg/g tissue)	17.3 ± 2.6	23.2 ± 2.9	23.5 ± 2.8	
Heart				
mass (%)	0.45 ± 0.03	0.47 ± 0.01	0.44 ± 0.01	

Eleven-week-old C57BL/6 male mice received water (CON), 20% sucrose solution (SUC), or sucrose solution plus 102 µmol phenelzine/kg body weight/day (SUC + PHE) to drink for 11 weeks. WAT, white adipose tissue; pWAT, perirenal WAT; eWAT, epididymal WAT; iWAT, inguinal WAT; BAT, brown adipose tissue; iBAT, interscapular BAT; TAG, triacylglycerol; Ac., accumulated. For accumulated intake of sucrose, chow, and energy, and energy efficiency, data are the mean ± SEM of 3 cages. A Student’s *t*-test was performed in these cases. The remaining data are the mean ± SEM of 8–12 animals per group. One-way ANOVA was followed by a Bonferroni post-hoc test. Difference CON vs. SUC mice: ^#^ *p* < 0.05; difference CON vs. SUC + PHE mice: ^§^ *p* < 0.05; ^§§^ *p* < 0.01.
